# Implementation and adoption of SOAP-M and SBAR at a German anesthesiology department – a single-center survey study

**DOI:** 10.1186/s12871-024-02650-2

**Published:** 2024-07-26

**Authors:** Oliver Keil, Justus Bernd Wegener, Benjamin Schiller, Mathäus Vetter, Markus Flentje, Hendrik Eismann

**Affiliations:** 1 Department of Anesthesiology and Intensive Care Medicine, Hannover Medical School, Carl- Neuberg-Str. 1, 30625 Hannover, Germany; 2 Business School, Middlesex University London, The Burroughs, London, NW4 4BT UK; 3 Department of Cardiology and Pulmonology, University Medical Center Goettingen, Robert- Koch-Straße 40, 37075 Goettingen, Germany

**Keywords:** Cognitive aids, SOAP-M, SBAR, Human factors, Patient safety

## Abstract

**Background:**

Checklists are a common tool used in order to mitigate risks caused by human factors and can facilitate the safe induction of anesthesia as well as handovers. SBAR (Situation, Background, Assessment, Recommendation) is a checklist recommended by the WHO and DGAI for handovers, while SOAP-M (Suction, Oxygen, Airway, Pharmaceuticals, Monitoring) is a checklist for the induction of anesthesia. This study investigates the implementation and adoption of these two checklists.

**Methods:**

We conducted a single-center online survey one year after the implementation of SOAP-M and SBAR at a university hospital’s anesthesiology department, using scales from three validated questionnaires to assess safety attitudes as well as the behavior of staff and the perceived usefulness of the checklists.

**Results:**

Staff with a high score in general attitude towards patient safety, as determined by the safety attitudes questionnaire, considered both checklists useful additions to their work environment. Nurses and physicians (*p* = 0.102) as well as groups divided according to work experience (*p* = 0.077) showed no significant differences in using SOAP-M and SBAR. Perceived usefulness was significantly higher (*p* < 0.001) among users of the checklists, and the same goes for positive reinforcement (*p* < 0.001), social cues (p *=* 0.0215) and goal cues (*p* = 0.0252).

**Conclusion:**

SOAP-M and SBAR are perceived as useful checklists for patient handovers and anesthesia induction by tertiary referral hospital’s employees with high score in general safety attitude and were therefore commonly used one year after their introduction. No significant difference in checklist adoption between occupations as well as groups divided according to work experience could be found. Perceived usefulness is significantly higher among users of the checklist, who feel using the checklists provides more support.

## Background

With around 7.3 deaths or serious complications caused by anesthetic procedures per million healthy patients (ASA I – II) undergoing elective procedures, anesthesia itself can be perceived as relatively safe [1]. But certain phases of an anesthetic procedure (e.g., induction of anesthesia, or handovers between providers) still pose significant risks for patients. Insufficient communication between providers contributes to the occurrence of these errors [2]. Checklists are a common tool used to reduce the rate of these events. Multiple publications have shown that checklists can reduce the rate of complications during anesthesia [3–6]. However, they nevertheless pose inherent risks, such as checklist fatigue, that may significantly reduce their effectiveness [5].

The surgical safety checklist (SSC) by the WHO [7] reduced perioperative mortality from 1.5 to 0.8% in 4,000 patients in 8 countries [2]. For anesthesia, the application of the WHO Checklist is not only recommended in the Helsinki Declaration on Patient Safety in Anaesthesiology by the European Society of Anesthesiology and Intensive Care [8], but also required for German anesthesiology departments by the German Federal Joint Committee (*Gemeinsamer Bundesausschuss*, G-BA) [9]. Nonetheless, this checklist has also been subject to criticism. For example, it has not consistently shown significant reduction in mortality in high-risk patients [10]. While the checklist might be sufficient from a surgical point of view, some aspects relevant to anesthesia that would contribute to close cooperation between physicians and nurses are not addressed [3]. These include planned airway access and fallback options, assessment of monitoring and preemptive checking for safety risks [5].

Staff at Hannover Medical School’s Department of Anesthesiology and Intensive Care Medicine formed a working group with a focus on perioperative communication to address these issues. Room for improvement was found in particular before the induction of anesthesia as well as during handovers between providers and departments (e.g., from anesthesia to intensive care). The working group quickly agreed that no further documented (paper or computer-based) checklist should be introduced, in order to increase motivation for actual usage.

The working group searched for a suitable checklist for the induction of anesthesia through an unstructured literature review, and found a suitable checklist: the pediatric sedation checklist by the American Academy of Pediatrics and the American Academy of Pediatric Dentistry [11]. Their acronym SOAPME was adapted to SOAP-M (**S**uction, **O**xygen, **A**irway, **P**harmaceuticals, **M**onitoring), and the checklist was adapted to the requirements of the department.

Since critical malfunctions of anesthetic equipment are rare, but are often overlooked in life-threatening situations, resulting in a high risk of morbidity and mortality for these patients [12], the working group wanted to highlight the importance of checking the equipment. Therefore, the item “Oxygen” also includes a QUICKcheck of the anesthesia workstation, including a verification of correct gas flow without leakage or obstruction, oxygen content in the gas flow and correct capnography function as recommended by the German Society of Anesthesia and Intensive Care (DGAI) [13]. A full version of the SOAP-M scheme can be found in the supplementary material. The department also introduced a pediatric version of SOAP-M, called pedSOAP-M, designed specifically for pediatric anesthesia, which was effective in detecting relevant errors during induction of anesthesia [14].

For patient handovers in a perioperative and intensive care setting as well es between providers, the briefing technique SBAR (**S**ituation, **B**ackground, **A**ssessment, **R**ecommendation) as recommended by the World Health Organization [15] as well as the DGAI was quickly identified as a suitable tool, and a German-language version as provided by the DGAI was adopted [16]. Both German and English versions of SOAP-M and SBAR can be found in the supplementary material.

SOAP-M and SBAR were introduced to the staff of the department, working in anesthesia and intensive a care, as a new briefing concept in a kick-off-event during the weekly mandatory training. Posters of both schemes were put up at all anesthetic workspaces, recovery wards and anesthetic intensive care units. All employees were also informed through an email, given a laminated pocket card and provided with an eLearning module. Employees were informed that these new tools are highly recommended by the working group as well as by the director of the department.

This study aims to assess the perceived usefulness as well as usage of the briefing concept using an online survey one year after its implementation. We consider this timeframe adequate for giving employees time to receive appropriate training, familiarize themselves with the concept, implement it into their work environment, and for fatigue to set in. Safety attitudes have repeatedly shown to influence checklist adoption [17, 18], and transfer climate is likely to have an impact [19]. We therefore assessed differences in these two variables between users and non-users of the briefing concept.

## Methods

### Data collection

We conducted an observational study, using an anonymous single-center online survey (Survey Monkey^®^, San Mateo, USA) one year after the implementation of SBAR and SOAP-M at Hannover Medical School’s Department of Anesthesiology and Intensive Care Medicine by the working group. The survey entailed sociodemographic questions (occupation, gender, relevant work experience), questions about the briefing concept, as well as selected scales from three questionnaires to keep the overall questionnaire concise and completable during routine work. The study was approved by the ethics committee of Hannover Medical School (8378_BO_K_2019).

### Survey instruments

Safety culture is an important variable in patient safety as well as in the implementation of cognitive aids [17, 18]. Though its definition is still not agreed upon [20], several instruments for its quantification have been developed. One of these is the safety attitudes questionnaire (SAQ). Increases in scores in this questionnaire have been associated with a reduction in complications in perioperative settings [21]. It is available in validated versions for different clinical surroundings (e.g., intensive care, operating rooms), each containing 30 core items [22], and has commonly been used for assessing cognitive aids in healthcare [23–25]. Zimmermann et al. [26] published a validation study on a translated German-language version of these items of the SAQ with satisfactory psychometric values, covering six dimensions of safety culture (teamwork climate, safety climate, job satisfaction, stress recognition, perception of management and working conditions). We used the SAQ to gain insight into the general attitude towards patient safety in the department.

The modified Training Evaluation Inventory (TEI) by Ritzmann et al. [27], originally published in german, is designed to assess the effectiveness of training and similar interventions. It contains ten scales, all of which achieved satisfactory reliability in the validation study. This study used the “perceived usefulness” scale to assess the perceived applicability of SOAP-M and SBAR to the intended work environment (level one of Kirkpatrick’s four levels of training evaluation [28]).

The Transfer Climate Questionnaire (TCQ) by Thayer and Theachout [29], translated and adapted by Hagemann et al. [30], is a common tool used to assess non-technical interventions in high-responsibility teams [19]. We applied the “goal cues”, “social cues”, “task cues”, “positive reinforcement” and “negative reinforcement” scales to assess the behavior of employees (level three of Kirkpatrick’s four levels of training evaluation [28]). All survey instruments used a five-point Likert scale from 1 = complete rejection to 5 = fullest approval.

### Statistical analysis

Statistical analysis was performed using IBM SPSS Statistics Version 28.0.1.0 as well as R Version 4.2.2 with R Studio Version 2022.12.0 + 353 [31]. Data is represented as mean ± standard deviation unless stated otherwise. Normal distribution was assessed for all scales using the Kolmogorov-Smirnov test, assuming normal distribution for *p* < 0.05. Several studies have reported insufficient internal validity of the “perception of management” subscale of the German-language version of the SAQ [23, 26]. Therefore, the Cronbach’s alpha or Spearman-Brown coefficient were calculated as applicable for all scales. Differences between occupations and groups divided according to relevant work experience were evaluated with t-tests and one-way ANOVA assuming statistical significance for *p* < 0.05. Two major occupational groups were defined: Physicians included all Residents and Consultants, while Nurses included anesthesia nurses, intensive care nurses, specialized anesthesia nurses, specialized intensive care nurses as well as anesthesia technicians, since they generally perform the same tasks in patient care in Germany. We also assessed the effect size for significant differences using Cohen’s *d* assuming small effect size for *d* > 0.2, medium effect size for *d* > 0.5 and large effect size for *d* > 0.8.

## Results

**Fig. 1:**
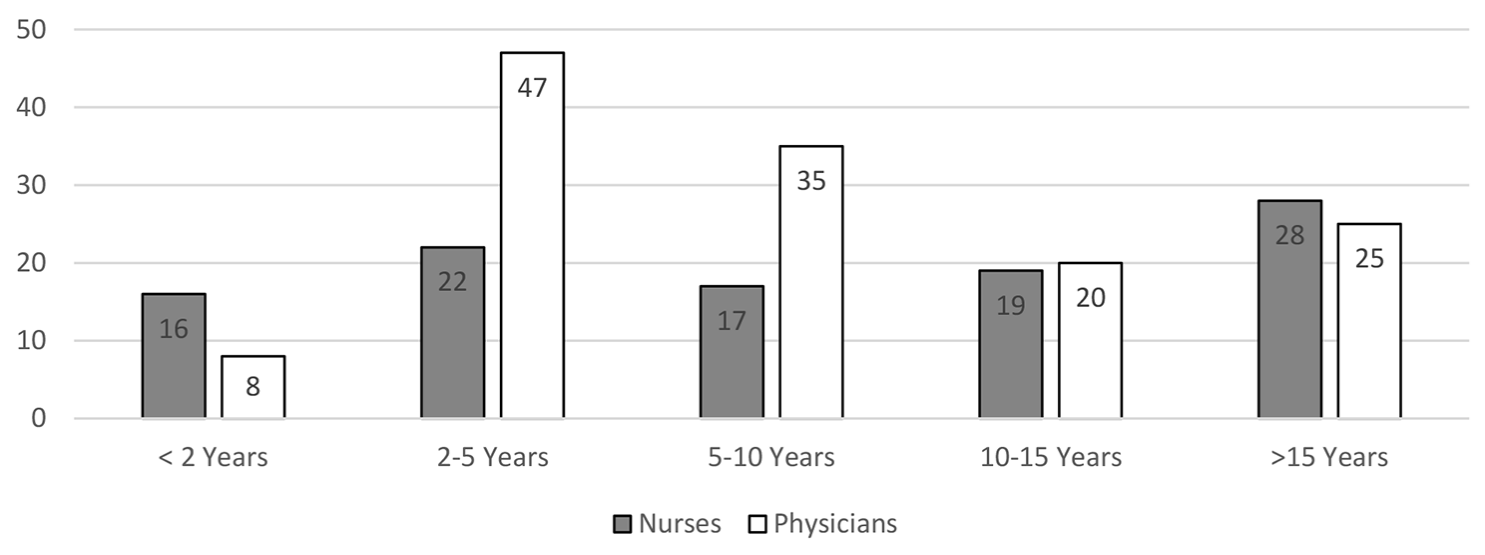
Relevant work experience by occupation

The survey was sent to all 379 employees (30 anesthesia technicians, 119 anesthesia nurses, 53 intensive care nurses, 177 intensive care and anesthesiology physicians) of Hannover Medical School’s Department of Anesthesiology and Intensive Care Medicine working in patient care. After the survey period (13th of May2019–23rd of May2019), 239 data sets from all relevant occupations (Table 1) and all levels of relevant work experience (Fig. 1) had been received (response rate 63.06%).

**Table 1:** Occupations of respondents

Occupation	*N* = 240	%
**Nurses**	**102**	**42.9**
Anesthesia nurses	41	17.1
Intensive care nurses	14	5.8
Specialized anesthesia nurses	22	9.6
Specialized intensive care nurses	6	2.5
Anesthesia technicians	19	7.9
**Physicians**	**138**	**57.5**
Resident currently in Anesthesia	50	20.8
Resident currently in Intensive Care	15	6.3
Consultant currently in Anesthesia	59	24.6
Consultant currently in Intensive Care	14	5.8

98.32% of all respondents were aware of information about SBAR & SOAP-M. While the pocket card, with 207 responses (80.5%), and the poster, with 196 responses (76.3%), reached most of the respondents, the kickoff-event reached 124 (51.67%). Only 21 (8.7%) respondents were aware of the eLearning module.

89.8% percent reported using SOAP-M and SBAR. Both the pocket card (with 56.36% of all users) and the posters (with 53.81% of all users) are commonly used, while 9.75% of all users use other non-disclosed mediums to include SOAP-M and SBAR in their clinical work. There were no significant differences with regards to use of SOAP-M and SBAR between nurses and physicians (*p* = 0.102), or between groups divided according to work experience (*p* = 0.077).

In line with other publications [23, 26], the “perception of management” subscale of the SAQ showed an insufficient Cronbach’s alpha of 0.179 (4 items), caused by a negative inter-item correlation of -0.596 for the item “management does not knowingly compromise the safety of patients”. We therefore excluded this item from further analysis, resulting in sufficient internal validity of all subscales and the SAQ itself (Table 2). All assessed scales achieved normal distribution in the Kolmogorov-Smirnov test, with *p* < 0.001.

**Table 2:** Safety attitudes Questionnaire

Scale	Cronbach’s alpha	Items	Total	User	Non User	*p*	Cohen’s d
**Teamwork Climate**	0.794	6	3.73 ± 0.46	3.75 ± 0.46	3.66 ± 0.51	0.4663	0.19
**Safety Climate**	0.849	7	3.52 ± 0.53	3.54 ± 0.53	3.42 ± 0.52	0.3159	0.23
**Job Satisfaction**	0.839	5	4.01 ± 0.68	4.05 ± 0.66	3.78 ± 0.77	0.1314	0.41
**Stress Recognition**	0.804	4	3.95 ± 0.86	3.97 ± 0.86	3.79 ± 0.91	0.4021	0.21
**Perception of Management**	0.179	3	3.36 ± 0.94	3.38 ± 0.95	3.25 ± 0.91	0.5576	0.13
**Working Conditions**	0.842	4	3.64 ± 0.99	3.69 ± 0.94	3.45 ± 1.19	0.3877	0.25
**SAQ**	0.914	29	3.71 ± 0.55	3.73 ± 0.54	3.56 ± 0.51	0.1755	0.32

**Fig. 2:**
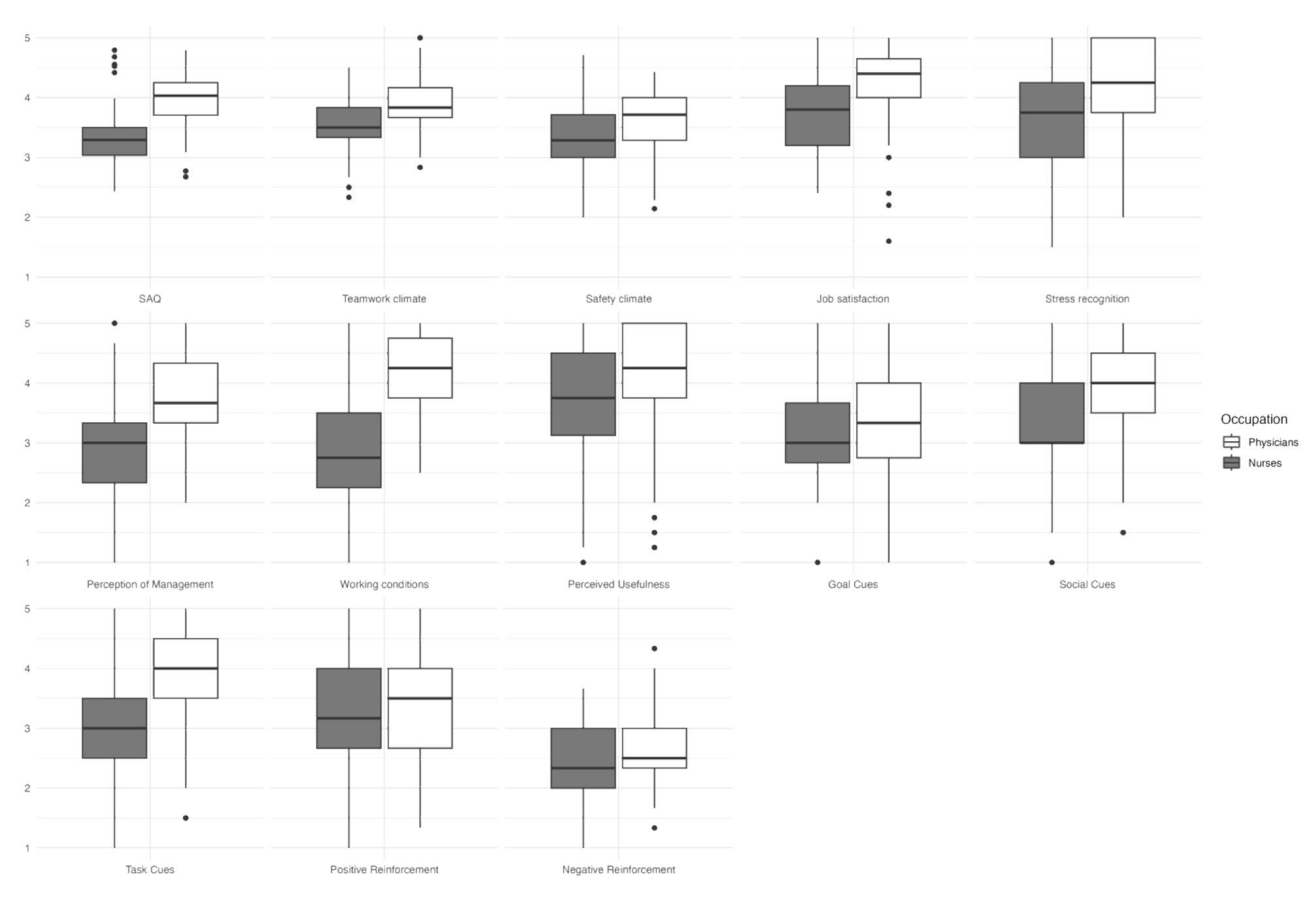
Box plots SAQ, TEI, TCQ

While the mean SAQ, as well as all of its subscales (Table 2), differ(s) significantly (*p* < 0.001, *d* = 1.53) between physicians (3.99 ± 0.41) and nurses (3.32 ± 0.48) (Fig. 2), no significant differences between groups divided according to relevant work experience (*p* = 0.096), or between users and non-users of SOAP-M and SBAR (Table 2), could be found in any assessed SAQ scales.

The “perceived usefulness” subscale of the TEI does not display significant differences between nurses (3.73 ± 1.04) and physicians (3.91 ± 1.17) (*p* = 0.2427), nor between groups divided according to work experience (*p* = 0.344), while there are significant differences between users and non-users of the checklists (Table 3). In line with other publications [19], the “negative reinforcement” scale from the Transfer Climate Questionnaire did not achieve satisfactory inter-item correlation. We therefore excluded this scale from further analysis. Out of those Transfer Climate Questionnaire scales that were used, the “goal cues”, “social cues” as well as “positive reinforcement” scales displayed significant differences between users and non-users (Table 3). The goal cues (*d* = 0.58) and social cues (*d* = 0.64) scales showed a medium effect size, while the “positive reinforcement” scale (*d* = 1.37) showed a large effect size. Only the “social cues” scale showed significant differences between groups divided according to work experience (p *=* 0.048), while the scales the “social cues” and “task cues” showed significant (*p* < 0.001) differences between nurses and physicians.

**Table 3:** Perceived usefulness, transfer climate questionnaire

Scale	Cronbach’s alpha	rho	Items	Total	User	Non-user	*p*	Cohen’s d
**Perceived Usefulness**	0.85	N/A	4	3.82 ± 1.12	3.93 ± 1.10	2.88 ± 0.95	< 0.001	0.98
**Goal Cues**	0.78	N/A	3	3.22 ± 0.84	3.27 ± 0.83	2.80 ± 0.83	0.0252	0.58
**Social Cues**	N/A	0.62	2	3.58 ± 0.89	3.64 ± 0.87	3.08 ± 0.87	0.0215	0.64
**Task Cues**	N/A	0.54	2	3.52 ± 0.97	3.58 ± 0.95	3.18 ± 1.04	0.1461	0.42
**Positive Reinforcement**	0.65	N/A	3	3.30 ± 0.82	3.39 ± 0.77	2.33 ± 0.75	< 0.001	1.37
**Negative Reinforcement**	0.21	N/A	3	2.52 ± 0.58	2.54 ± 0.57	2.27 ± 0.61	0.0987	0.47

N/A = Not applicable

## Discussion

Our study shows that SOAP-M and SBAR are perceived as useful checklists for patient handovers and anesthesia induction by university hospital employees with a good general safety attitude, and they were therefore commonly used one year after their introduction. There are no significant differences in the adoption of the checklists between occupations nor between groups divided according to work experience. The usefulness is rated significantly more highly by users of the checklist, who feel using the checklists provides them with more support.

We consider the response rate of 63.06% to be satisfactory for this non-mandatory online questionnaire. One year after the implementation, 89.8% percent of responders reported using these tools, with both the pocket card (with 56.36% of all users) and the posters (with 53.81% of all users) apparently being used frequently. Other publications showed similar adoption rates during simulation [32], and lower rates for emergencies [17], indicating a high degree of overall adoption of the briefing concept. Thus, we regard the implementation and adoption of SOAP-M and SBAR to most likely be adequate during the survey period. Since some users use both tools, investigations into their reasoning for using one over the other might be of interest. Surprisingly, the posters and pocket cards reached most employees, while only 8.7% of responders were aware of the eLearning module. Of these three means of distribution, the production of the eLearning module required by far the most financial and human resources. Since all employees regularly receive simulation training highlighting the importance of cognitive aids, introduction of cognitive aids that are used multiple times during any given shift might be feasible during routine simulation training paired with a kickoff event. We therefore conclude that the additional production of expensive training tools like eLearning modules should only be initiated after careful consideration and might be not necessary in organizations with good general safety attitudes and high positive reinforcement scores. Posters and pocket cards seem to achieve sufficient coverage and sufficient perceived knowledge for nurses and physicians to adopt these specific cognitive aids in these environments. Since both posters and pocket cards require relatively little financial and especially human resources, we theorize that both should be included in a structured approach to cognitive aid implementation.

Employees consider the introduction of SOAP-M and SBAR a useful addition to their work environment, rating the perceived usefulness as 3.82 ± 1.12 on a five-point Likert scale. No significant differences regarding the use of these checklists could be found between groups divided according to occupation or work experience, which indicates sufficient adoption of both tools in the work environments of employees regardless of their occupation or work experience. We therefore theorize that staff members have a sense of responsibility for patients, regardless of their occupation or work experience. This might be a result of firmly established team simulation training in the surveyed department.

While general safety attitudes, with a mean SAQ of 3.71 ± 0.55 on a five-point Likert scale, can generally be considered good, we found significant differences between nurses and physicians in all subscales, in line with other publications [23, 33]. The origin of these differences remains unclear and cannot be elucidated in this study. In general, cognitive aid implementation is understood to be interdependent with employees’ safety attitudes [17, 18]. Therefore, differences in cognitive aid adoption between working groups could be expected. This study, however, showed no statistically significant differences in the likelihood of using SOAP-M and SBAR between nurses and physicians (*p* = 0.102) and groups divided according to work experience (*p* = 0.077). We therefore argue that, although differences in assessments of safety attitudes might be measurable between groups, they do not have to result in differences regarding checklist adoption, especially since no significant differences in the SAQ were found between users and non-users of the checklists.

Since both nurses (3.73 ± 1.04) and physicians (3.91 ± 1.17) rate the perceived usefulness relatively high on a five-point Likert scale, we consider both tools likely to be applicable to the work environment of both relevant occupational groups. Furthermore, no significant differences between groups divided according to work experience were found, which indicates that the checklists are applicable regardless of work experience.

Users of the checklists reported significantly higher scores with regards to goal cues, social cues as well as positive reinforcement. While the differences relating to goal cues and social cues only showed a medium effect size, the differences in positive reinforcement were not only significant, but also had a large effect size. We therefore perceive positive reinforcement experienced by employees to be a facilitator for checklist adoption in this study. As already shown in other studies, leadership support is associated with successful checklist implementation [34]. We think our data furthermore suggests that individual checklist adoption is also interlinked with social interactions regarding checklist use perceived by individual employees.

We recognize several limitations of this study. First and foremost, this study did not observe the applicability and use of SOAP-M and SBAR on the job, but through questionnaires. Actual applicability and use of these tools might differ considerably from the attained measurements. Due to its monocentric design and single survey period, the results of this study might not only be inapplicable to other anesthesiology departments, but also be insufficient to evaluate the applicability of the tools as well as the behavior of employees in the long run. Furthermore, employees that did not adopt the checklists might have participated significantly less than users, resulting in a skewed assessment of the adoption even at the chosen time of one year after the implementation. Furthermore, different cognitive aids might have been used by providers during the survey period, that might have influenced the questionnaire results.

## Conclusion

This study likely demonstrates the adequate adoption of two perioperative checklists (SOAP-M and SBAR) one year after their implementation. This was achieved through a kickoff event, posters, pocket cards and an eLearning module in a university hospital’s anesthesiology department. Both checklists were implemented in a setting with high scores in attitudes towards patient safety, as determined by the safety attitudes questionnaire, and are perceived as useful additions to the work environment by employees, and thus are commonly used. No significant differences in checklist adoption between occupations as well as groups divided according to work experience were found in this study, implying that the checklists are applicable regardless of experience or occupation. Users of the checklist report not only a significantly higher perceived usefulness, but also significantly higher scores for positive reinforcement as well as goal cues and social cues due to using the checklists, although all responses came from the same work environment.

## Data Availability

The data sets used and/or analyzed during the current study are available from the corresponding author on reasonable request.
